# Development of autonomic heart rate modulations during childhood and adolescence

**DOI:** 10.1007/s00424-024-02979-0

**Published:** 2024-06-28

**Authors:** Kateřina Helánová, Martina Šišáková, Katerina Hnatkova, Tomáš Novotný, Irena Andršová, Marek Malik

**Affiliations:** 1 Department of Internal Medicine and Cardiology, University Hospital Brno, Jihlavská 20, 625 00 Brno, Czech Republic; 2 Department of Internal Medicine and Cardiology, Faculty of Medicine, Masaryk University, Jihlavská 20, 625 00 Brno, Czech Republic; 3 National Heart and Lung Institute, Imperial College, 72 Du Cane Rd, Shepherd’s Bush, London, W12 0NN England

**Keywords:** Heart rate, Heart rate modulations, Children, Age-dependency, Sympatho-vagal balance

## Abstract

Autonomic control of heart rate is well known in adult subjects, but limited data are available on the development of the heart rate control during childhood and adolescence. Continuous 12-lead electrocardiograms were recorded in 1045 healthy children and adolescents (550 females) aged 4 to 19 years during postural manoeuvres involving repeated 10-min supine, unsupported sitting, and unsupported standing positions. In each position, heart rate was measured, and heart rate variability indices were evaluated (SDNN, RMSSD, and high (HF) and low (LF) frequency components were obtained). Quasi-normalized HF frequency components were defined as qnHF = HF/(HF + LF). These measurements were, among others, related to age using linear regressions. In supine position, heart rate decreases per year of age were significant in both sexes but lower in females than in males. In standing position, these decreases per year of age were substantially lowered. RMSSD and qnHF indices were independent of age in supine position but significantly decreased with age in sitting and standing positions. Correspondingly, LF/HF proportions showed steep increases with age in sitting and standing positions but not in the supine position. The study suggests that baseline supine parasympathetic influence shows little developmental changes during childhood and adolescence but that in young children, sympathetic branch is less responsive to vagal influence. While vagal influences modulate cardiac periods in young and older children equally, they are less able to suppress the sympathetic influence in younger children.

**Supplementary Information:**

The online version contains supplementary material available at 10.1007/s00424-024-02979-0.

## Introduction

Autonomic control of cardiac sinus nodal periodicity is well known and has repeatedly been studied in adults [17, 20, 66]. Left-sided vagus nerve has direct influence on the sinus node [30] and physiologic parasympathetic tone decreases the cardiac periodicity from the basal heart rate of 110–120 beats per minute (bpm) to the usual physiologic rate of 50–70 bpm [44]. Efferent sympathetic nerve fibres counter the vagal effects and accelerate heart rate by increasing the rate of sinus nodal depolarizations [20, 63]. It is also well known that both limbs of the autonomic nervous system react with different lag time [35, 36]. Although parasympathetic effects can occur within a single cardiac period, vagal heart rate modulations standardly respond, among others, to the respiration-induced changes in the intra-thoracic pressure and lead to respiratory arrhythmia [58]. On the contrary, sympathetic system reacts more slowly and takes 10 to 20 s to respond to control requirements [20]. This creates the basis for the assessment of the proportions between vagal and sympathetic heart rate modulations based on spectral analysis of heart period series [47, 62].

This all has been well documented in adults. The situation is somewhat different in children in whom the physiologic resting heart rate is approximately 120–130 bpm at birth and subsequently decreases and reaches the adult values at late puberty [61, 62]. The physiologic processes responsible for this heart rate development in children are only approximately understood. Studies investigating distinction between the vagal and sympathetic reactions in children are sparse. A recent study used limited measurements of heart rate variability (HRV) in large paediatric populations and proposed a gradual age-related increase in the vagal cardiac influence with differences between sexes. Linear decrease of sympathetic influence with age has also been suggested [21]. Nevertheless, while numerous investigations researching spectral HRV analysis in children have been reported [13, 25, 41, 50, 62], little data exist on paediatric heart rate responses and spectral HRV analyses based on controlled provocations that can be expected not only to involve autonomic nervous system reflexes and reactions but also to distinguish between different stages of sympatho/vagal balance.

To address these data gaps as well as to contribute to the knowledge of the development of heart rate reactions to specific provocations in paediatric subjects, we report heart rate and spectral HRV analyses in a sizeable population of children and adolescents who were investigated during a series of strictly controlled repeated postural provocations.

## Methods

### Study population

We collected the data from a population of school-aged children and adolescents. The design of the study aimed at reaching uniform age distribution, both in females and males, between the ages of 4 and 19 years. The study enrolment was organized at preparatory, primary, secondary, and up to pre-university schools in the Czech Republic, specifically in central Moravia and southern Silesia regions. As a byproduct of the study, we offered all participants health checks including the assessment and diagnosis of electrocardiogram (ECG) recordings. The Ethics Committee of the University Hospital Brno reviewed the study protocol and approved the study conduct. The study strictly adhered to the principles of the World Medical Association’s Declaration of Helsinki. Written participation consent was provided by all participants who were, at the time of enrolment, of legal capacity age according to the local law. For other participants, the consent was provided by their parents or legal guardians. The consent included clauses of research utilization of anonymized data as well as of demographic data collection.

### Postural provocations and ECG recordings

SEER MC version 2 ECG recorders (GE Healthcare, Milwaukee, WI) were used in the study. The recorders provided 12-lead ECG signals sampled at 1 kHz and were used with electrodes in the Mason-Likar positions. In each participant, continuous ECG was recorded during a 70-min provocative protocol that consisted of a series of body position changes. Specifically, the participants started in strict supine position, followed by sitting, standing, supine, standing, sitting, and supine positions that were adopted in this order. Each position was maintained, per protocol, for 10 min while the changes between the positions were required within 20 s. The postural positions were maintained without any unnecessary movements. In the sitting and standing positions, study subjects did not have any back support (i.e., sitting was performed on benches without any backrest, standing was performed without touching walls or any other support).

The study was conducted in groups of subjects of similar ages who performed the postural manoeuvres at the same time. Groups of up to 20 subjects were investigated and the investigations were conducted indoors (e.g., school sports halls) always in the mid-morning hours. For each investigated group, the timings of the position changes were recorded. Age-appropriate fiction tales without any exciting contents were read to small children to keep them focused on the protocol requirements. All others were investigated in a noise-free environment without any external disturbances. The participants were not allowed to speak and/or non-verbally communicate during the investigations.

Prior to the investigations, standard school activities of the morning hours (but without any sport or physical exercise) were followed by all participants. During the study days, none of the participants smoked or consumed caffeinated or energy drinks prior to the study conduct. For practical reasons, the ECG recordings were started before the study protocol separately in different children (and also separately disconnected). The timings of the start and end of the recordings were recorded allowing to identify the individual postural positions accurately within the periods of the recorded signals.

### QRS detection

For the purposes of ECG computer processing, continuous digital 12-lead ECG recordings were divided into 10-s segments which were shifted by 5 s leading to a 5-s overlap between subsequent segments. Previously described software routines [23, 37] were used to process each segment and the overlaps between neighbouring segments ensured that no QRS complex was missed when occurring at the edge of a segment. In each segment, QRS complexes were localized by 4 different algorithms that were based on different signal processing principles [10, 27, 28, 43]. When the results of the algorithms were mutually consistent, the detected positions of the QRS complexes were used in subsequent analyses. Otherwise, the 10-s ECG segment was reviewed visually and, where appropriate, the sequence of QRS positions was edited manually. The operators performing the ECG review had no access to the timing of the 10-s segments within the study protocol.

### Heart rate and heart rate variability

Linking the QRS detections in separate 10-s ECG segments provided a complete RR interval stream for each subject and for each postural position. To eliminate transient heart rate changes around position changes, we eliminated the first and last minute of each period which provided central 8 min of RR interval data for each postural position. Among these 8-min data, all 5-min subintervals were identified which contained no ectopic beats. If no 5-min subinterval free of ectopic beats was found, the postural position of the given subject was excluded from the analysis. In other cases, the 5-min sub-interval was found that showed the most stable heart rate. This was assessed by computing the slope of linear regression between RR interval durations and the central time moments of the RR intervals. The 5-min subinterval with the lowest absolute value of this slope was selected.

Heart rate was measured in the selected 5-min subintervals by calculating the average RR interval duration and converting it into rate expressed in bpm.

Simple time-domain HRV indices were obtained from the RR sequences of the selected 5-min subintervals and included two values [62]:

- SDNN – standard deviation of all RR intervals, and
- RMSSD – root mean square of the differences between successive RR intervals.

SDNN represents total variability of cardiac periods with no distinction between sympathetic and vagal influences. RMSSD expresses very short-term variations of cardiac periods and, under stable conditions, is mainly, although not entirely, influenced by respiratory arrhythmia. It might thus be taken as an approximate manifestation of vagal modulations.

Subsequently, the RR interval sequences of the selected 5-min subintervals were cubic spline interpolated to obtain discrete signals with a constant frequency of 1 kHz and with floating point RR interval values. These continuous RR interval signals were processed by Blackman-Tukey modification of Fast Fourier transformation with Hann window [24] to obtain spectral HRV characteristics [62]:

- LF – low frequency components, i.e., the frequency power within the spectral band of 0.04–0.15 Hz,
- HF – high frequency components, i.e., the frequency power within the spectral band of 0.15–0.40 Hz,
- LF/HF ratio – obtained as a proportion between absolute values of the LF and HF components,
- Quasi-normalized HF components expressed as qnHF = HF/(LF + HF).

While LF components represent a mixture of sympathetic and vagal modulations, HF components are associated with vagal influence of RR periodicity [62]. Nevertheless, direct comparison of LF and HF components measured in different recordings might be problematic since the numerical values of both components depend on the underlying heart rate [35, 36]. For that reason, the LF/HF ratios and the quasi-normalized HF components were primarily used in the analyses of the study as their technical dependence on heart rate is less pronounced. The LF/HF ratios and quasi-normalized HF components are non-linearly reciprocal and, since both are obtained from HF and LF components, each can be derived from the other. The non-linear reciprocal relationship is the basis for the usual interpretation of the indices. Increases in LF/HF ratio might be interpreted as a surge in sympathetic modulations, increases in qnHF components indicate augmentation of vagal effects on RR variations. Quasi-normalization may also be applied to the LF components but has not been used because qnLF = LF/(LF + HF) only numerically complements qnHF (since qnLF + qnHF = 1) and thus does not provide any additional information or alternative interpretation to the LF/HF ratio. The quasi-normalized components have been derived as approximation of standard normalized HF and LF components that are derived from autoregressive spectral analysis [40, 62]. The autoregressive spectral analysis was not used in this study since it is not obvious whether the same setting of autoregressive models would be valid for data of differently aged children. Indeed, differences in spectral estimates by different methods of HRV analysis applied to paediatric data have previously been described [46].

In each study subject, the heart rate values and HRV indices obtained during corresponding postural positions were averaged. In this way, characterizations of supine, unsupported sitting, and unsupported standing positions were obtained for individual subjects.

## Statistics and data presentation

### Continuous data are presented as mean ± standard deviation

Heart rate and HRV indices were related to the age of study subjects (separately for both sexes) using linear regression analyses. Regression lines were constructed together with their 95% confidence intervals and presented graphically. The regression slopes were tested against zero using Student’s t distribution.

Different age-limited sub-groups of the study population were defined by a window of ± 1 year around a central age of the sub-group. In such sub-groups, means and standard errors of heart rate and HRV indices were graphically displayed and overlayed for female and male subjects. This allowed visual approximation of statistical significances of sex differences as well as between non-overlapping population sub-groups. (The larger the gap between ± standard errors or means, the stronger the statistical significance.)

For the purposes of exact statistical evaluation, the study population was divided into approximate age-related quartiles. In each of these quartiles, heart rate and HRV indices were compared between female and male subjects using two-sample two-tail t-test. Differences in the variances of the compared values were assumed. Differences between the quartiles were tested in each sex separately. One-way ANOVA with Tukey post hoc test was used for this purpose. P-values < 0.05 were considered statistically significant. No p-value threshold adjustment for multiplicity of testing was used since the compared data in different tests were mutually interlinked. Abbreviation NS is used to indicate statistically non-significant results of the tests. Statistical tests were omitted in some cases in which the difference was obvious from graphical displays.

IBM SPSS Statistics package (version 29.0.0.0) was used in the statistical calculations. Signal processing of the ECG signals and the heart rate and HRV calculations were performed using a previously validated in-house software packages [24] that were implemented in C/C +  + (Microsoft Visual Studio 2022, version 17.4.3).

## Results

### Population and electrocardiographic data

**Fig. 1:**
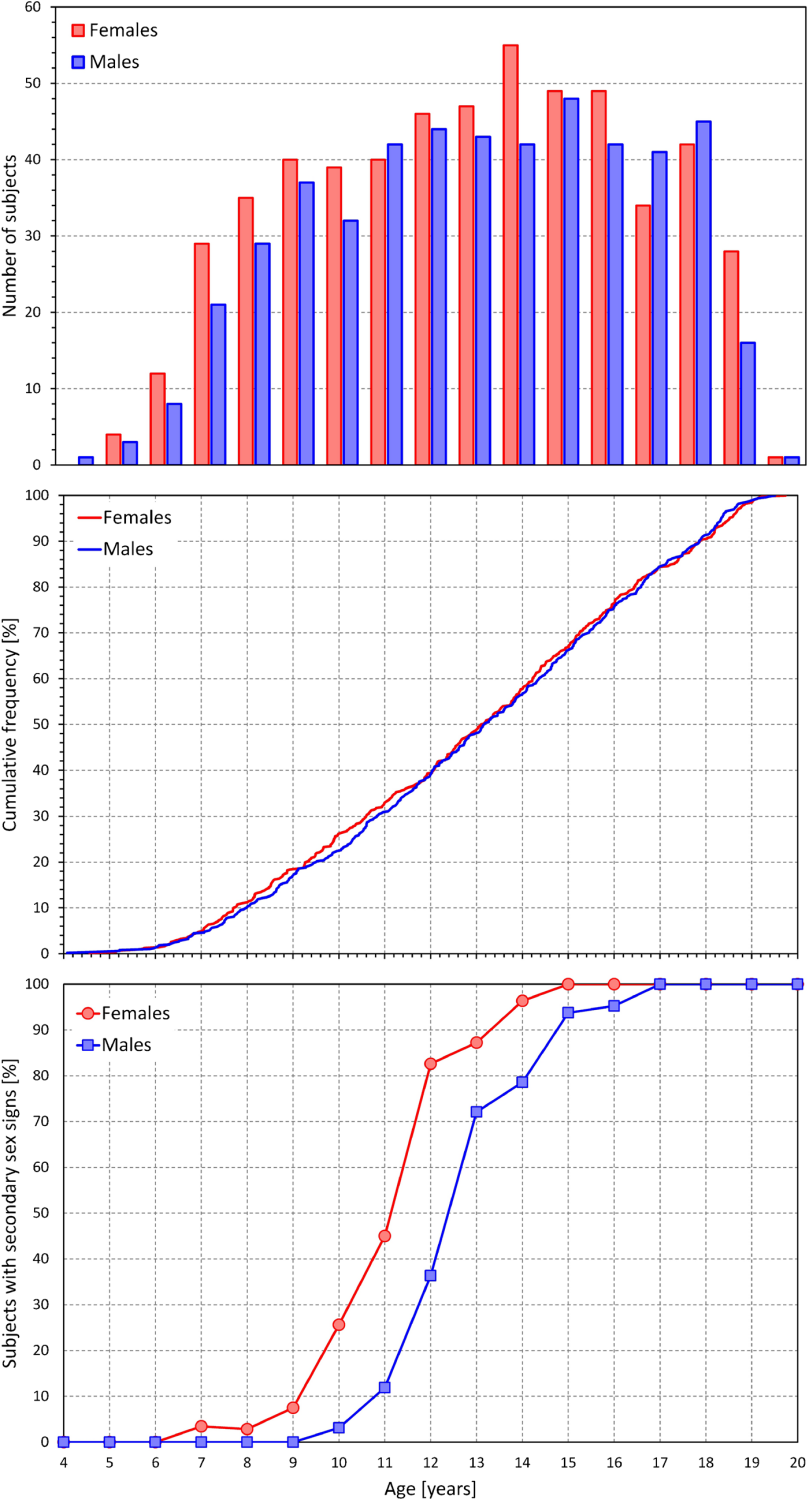
Figure shows the composite of the study population. The top panel shows the number of subjects in different age groups (age of each subject rounded to the closest integer), the middle panel shows cumulative distributions of the exact ages, the bottom panel shows the cumulative distribution of subjects who showed secondary sex signs. Red and blue bars, marks and lines correspond to female and male subjects, respectively

The investigation was performed in 1094 subjects. Of these, 49 (4.5%) were subsequently excluded because of being on drugs potentially affecting cardiac electrophysiology or on hormonal contraceptives, having a cardiac abnormality or a history of corrected cardiac abnormality, or undergoing sex transversal procedures. The study population thus consisted of 1045 subjects who were all healthy according to clinical investigation performed at enrolment. Of these subjects, 550 were females (aged 12.92 ± 3.64 years) and 495 were males (aged 13.03 ± 3.56 years). No significant difference between the ages of female and male participants was found. The age distribution of the study population is shown in the top panel of Fig. 1. The middle part of the figure shows that between ages of 6 and 19 years, the age distribution was practically uniform in both sexes. This distribution also shows that it was reasonable to approximate the age-related quartiles of the population using age dichotomies of 10, 13, and 16 years. Specifically, in the age groups below 10 years, 10 to 13 years, 13 to 16 years, and above 16 years, there were 144 and 111, 124 and 127, 152 and 136, and 130 and 121 females and males, respectively.

Figure 1 (bottom panel) also shows the development of secondary sex signs in study subjects. In girls, the secondary sex signs appeared around a median of 11 years of age while in boys, the signs emerged some 1.5 years later.

Among the study subjects, 15 females and 11 males did not complete the full provocative protocol. During the investigation, they suffered from physical discomfort, pre-syncopal symptoms, or nausea. Occasional vomiting occurred in small children. In these subjects, partial data of the postural positions completed before the occurrence of side-effects were included in the analyses.

## Heart rates

**Fig. 2:**
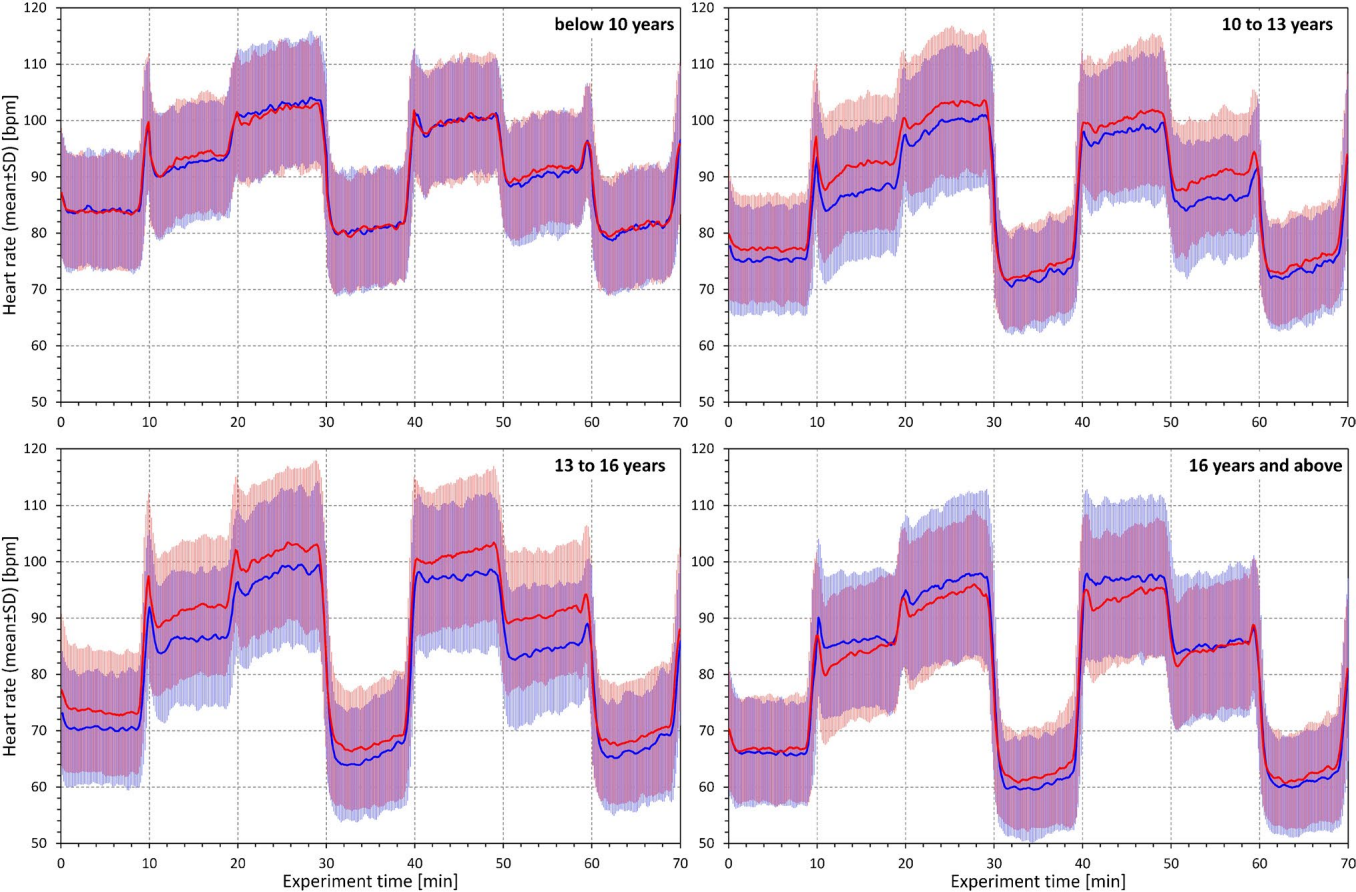
Heart rates in beats per minute as measured during the 7 phases of the study (the distinction of the 10-min phases is well visible in figure panels: supine → sitting → standing → supine → standing → sitting → supine). In each panel, the red and blue bold lines show mean values in females and males respectively, the red and blue bands show mean ± standard deviation in females and males respectively, the violet bands show the overlap between the mean ± standard deviation bands of both sexes. The four panels correspond to the age groups as indicated: below 10 years, 10 to 13 years, 13 to 16 years, and above 16 years

**Fig. 3:**
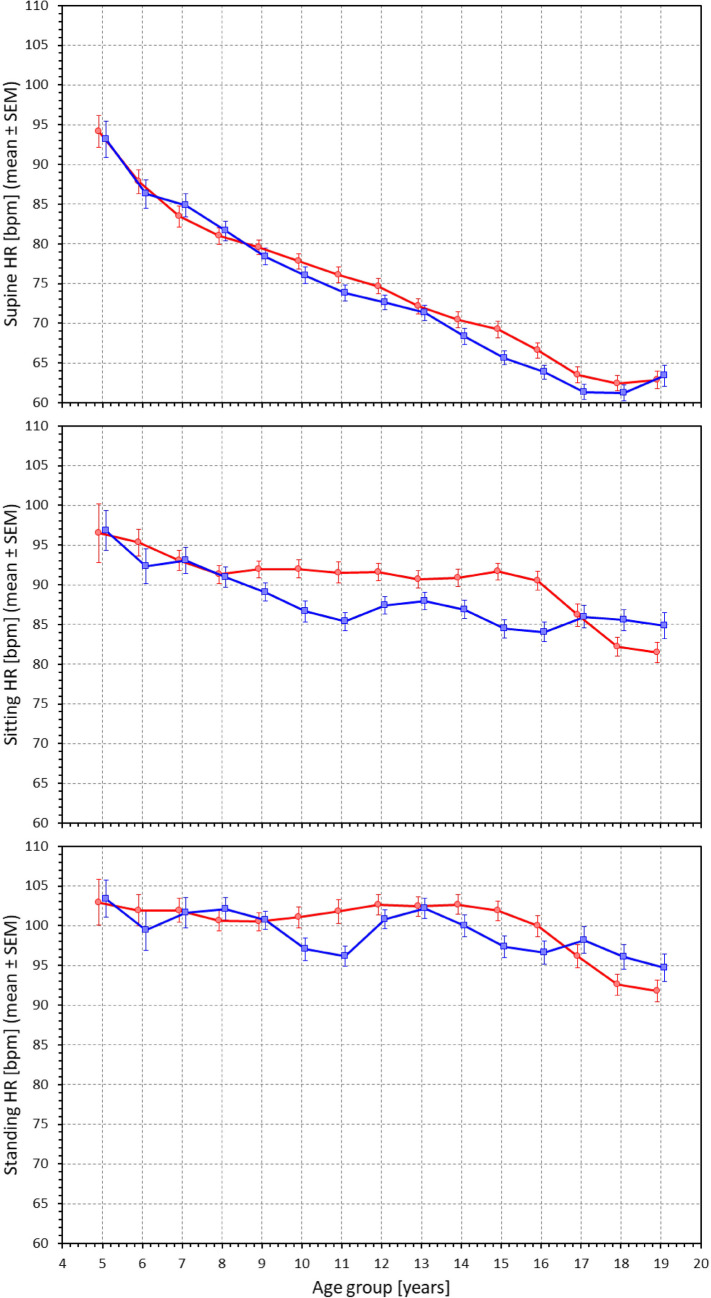
Heart rates as measured in different 2-year bins of the population. For each age 5 to 19 years, the figure shows heart rate values (mean ± standard errors of mean) in the sub-population aged ± 1 year of the age indicated (i.e., at age 5, the mean of subjects aged 4 to 6 years is shown). The top, middle, and bottom panel correspond to supine, sitting, and standing positions, respectively. Red circles and red lines correspond to female subjects, blue squares and blue lines to male subjects. HR – heart rate, SEM – standard error of mean

**Fig. 4:**
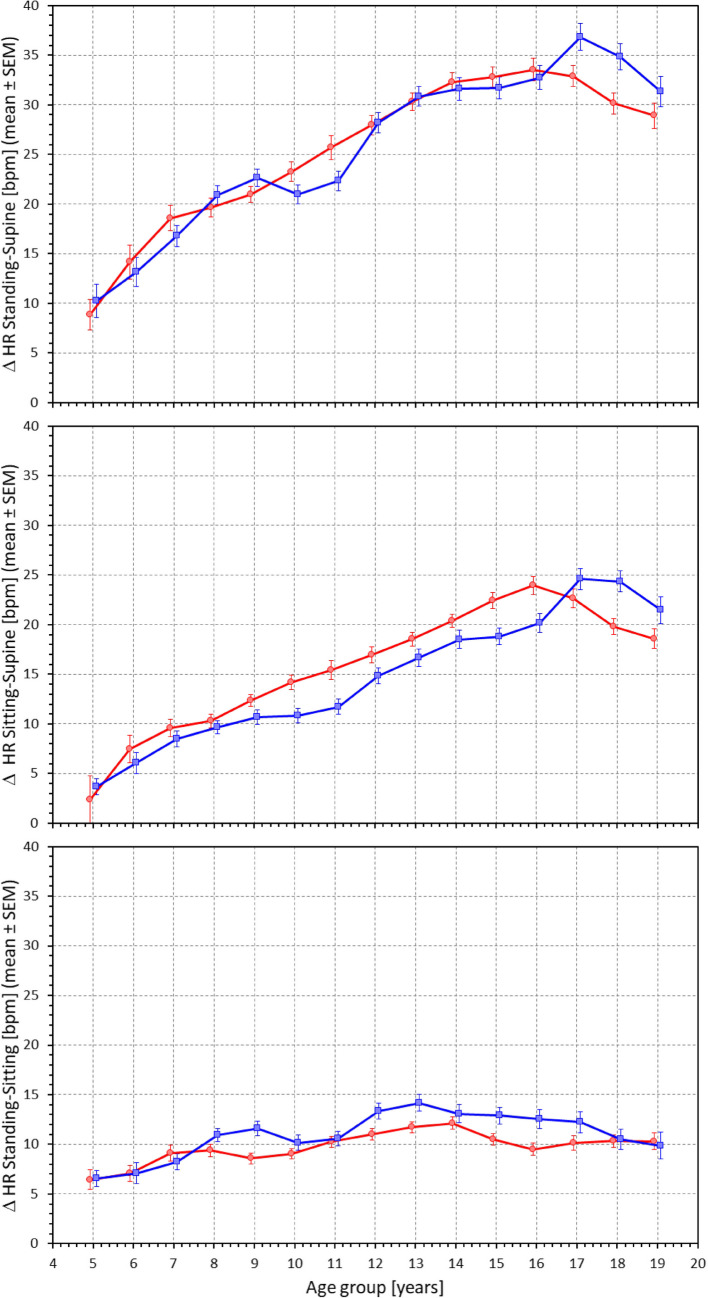
Differences between heart rates measured in standing and supine positions (top panel), sitting and supine positions (middle panel) and standing and sitting positions (bottom panel). The layout of the figure, definition of population strata, and distinction between females and males are the same as in Fig. 3. Δ HR – heart rate differences, SEM – standard error of mean

Heart rate data in the study quartiles are shown in Fig. 2 and statistically summarized in Table 1. As expected, the supine heart rate progressively decreased with increasing age (p < 0.0001 in both sexes). A similar decrease with age was also seen in sitting and standing heart rates but was numerically much less pronounced. This is well visible in Fig. 3 which shows supine, sitting, and standing heart rates in population sub-groups 4–6, 5–7, etc., up to 18–20 years. The heart rate differences between postural positions are shown in Fig. 4 which shows that the effect of postural changes on heart rate was much smaller in little children than in adolescents.

**Table 1:** Heart rate values

	Female	Male	p-value (F vs M)	p-value (age groups)
**Position 1: Supine**				
Age < 10 years	83.62 ± 9.73	83.78 ± 9.91	NS	F: < 0.0001 M: < 0.0001
Age 10–13 years	77.11 ± 9.20	75.28 ± 9.20	NS	
Age 13–16 years	73.22 ± 10.7	70.16 ± 9.49	0.0054	
Age > 16 years	66.33 ± 9.16	65.78 ± 8.84	NS	
**Position 2: Sitting**				
Age < 10 years	93.60 ± 9.76	92.14 ± 9.97	NS	F: < 0.0001 M: < 0.0001
Age 10–13 years	92.09 ± 11.60	87.31 ± 11.04	0.0005	
Age 13–16 years	91.57 ± 11.91	85.98 ± 11.81	< 0.0001	
Age > 16 years	83.69 ± 12.04	85.85 ± 11.72	NS	
**Position 3: Standing**				
Age < 10 years	102.20 ± 10.80	102.63 ± 11.15	NS	F: < 0.0001 M: < 0.0057
Age 10–13 years	103.16 ± 12.46	99.71 ± 12.45	0.0154	
Age 13–16 years	102.03 ± 13.22	98.85 ± 13.45	0.0237	
Age > 16 years	94.65 ± 11.92	96.60 ± 14.11	NS	
**Position 4: Supine**				
Age < 10 years	80.57 ± 9.90	80.20 ± 9.90	NS	F: < 0.0001 M: < 0.0001
Age 10–13 years	73.10 ± 8.45	71.69 ± 9.08	NS	
Age 13–16 years	66.92 ± 10.73	64.39 ± 9.74	0.0214	
Age > 16 years	61.50 ± 8.65	59.90 ± 8.65	NS	
**Position 5: Standing**				
Age < 10 years	100.14 ± 10.81	100.09 ± 9.87	NS	F: < 0.0001 M: NS
Age 10–13 years	100.48 ± 12.40	98.18 ± 11.79	NS	
Age 13–16 years	101.54 ± 12.72	97.46 ± 13.10	0.0045	
Age > 16 years	93.94 ± 11.44	96.97 ± 13.74	0.0314	
**Position 6: Sitting**				
Age < 10 years	91.41 ± 9.67	90.32 ± 10.40	NS	F: < 0.0001 M: < 0.0004
Age 10–13 years	90.45 ± 10.16	85.90 ± 10.64	0.0003	
Age 13–16 years	90.55 ± 11.22	84.46 ± 10.77	< 0.0001	
Age > 16 years	84.76 ± 11.19	85.12 ± 12.46	NS	
**Position 7: Supine**				
Age < 10 years	80.92 ± 9.94	80.69 ± 9.69	NS	F: < 0.0001 M: < 0.0001
Age 10–13 years	74.68 ± 8.85	73.11 ± 9.15	NS	
Age 13–16 years	68.17 ± 10.30	65.84 ± 9.31	0.0251	
Age > 16 years	61.53 ± 8.50	60.43 ± 8.60	NS	

Heart rate values shown (mean ± standard deviation) in beats per minute. P-values “F vs M” show statistical comparison of both sexes, “age groups” show inter-sex comparisons of age-quartiles. F – females, M – males, NS – non significant

**Fig. 5:**
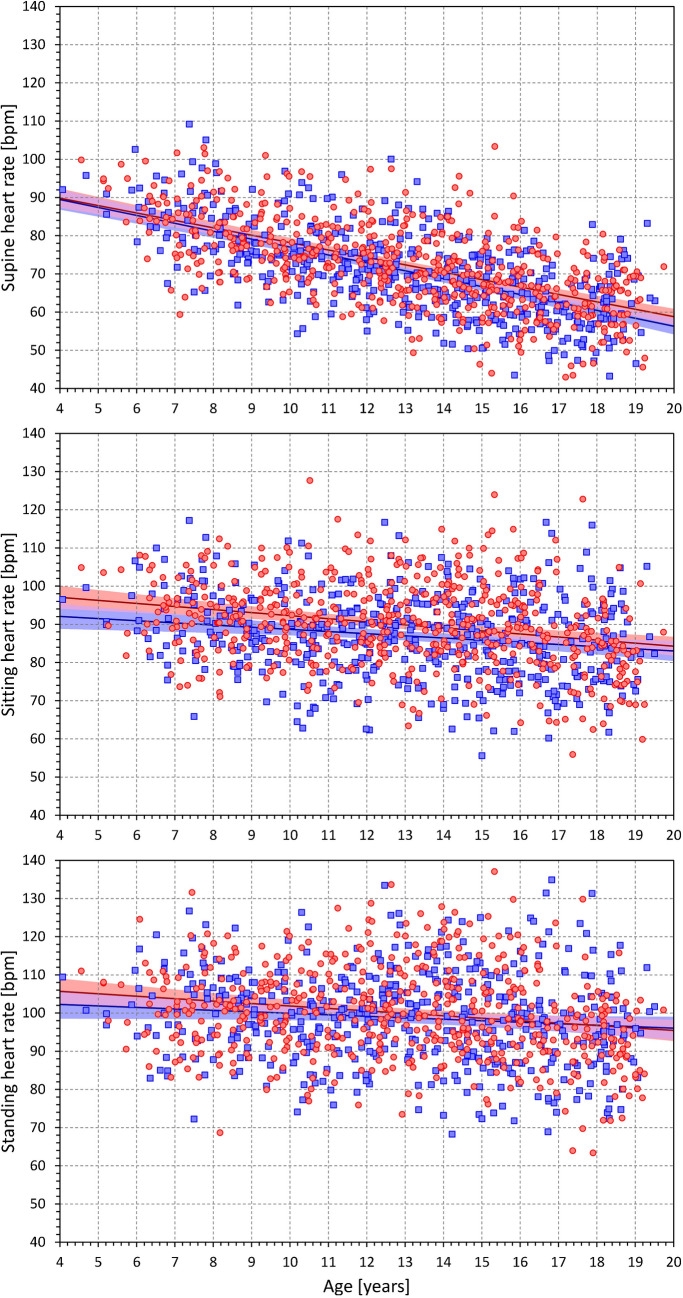
Scatter diagrams between the ages of study subjects and their heart rates measured in supine position (top panel), sitting position (middle panel) and standing positions (bottom panel). In each panel, the red circles and blue squares correspond to female and male participants, the red and blue bold lines are linear regression between the displayed values and the age of the subjects, the light red and light blue bands are 95% confidence intervals of the linear regressions. The violet bands are the overlaps between the confidence intervals of both sexes

The same age development can be seen in Fig. 5 which shows regression slopes between heart rate and age gradually decreasing from supine to standing positions. In supine position, the heart rate decreased by 1.94 and 2.07 bpm per year of age in females and males, respectively (p < 0.0001 for both sexes). In sitting and standing positions, the corresponding slope values were 0.80 and 0.57, and 0.64 and 0.39 bpm/year, respectively. All these slopes were statistically significant with p < 0.0001; only in males on standing, the significance of the slope was reduced to p = 0.0133.

## Heart rate variability

**Fig. 6:**
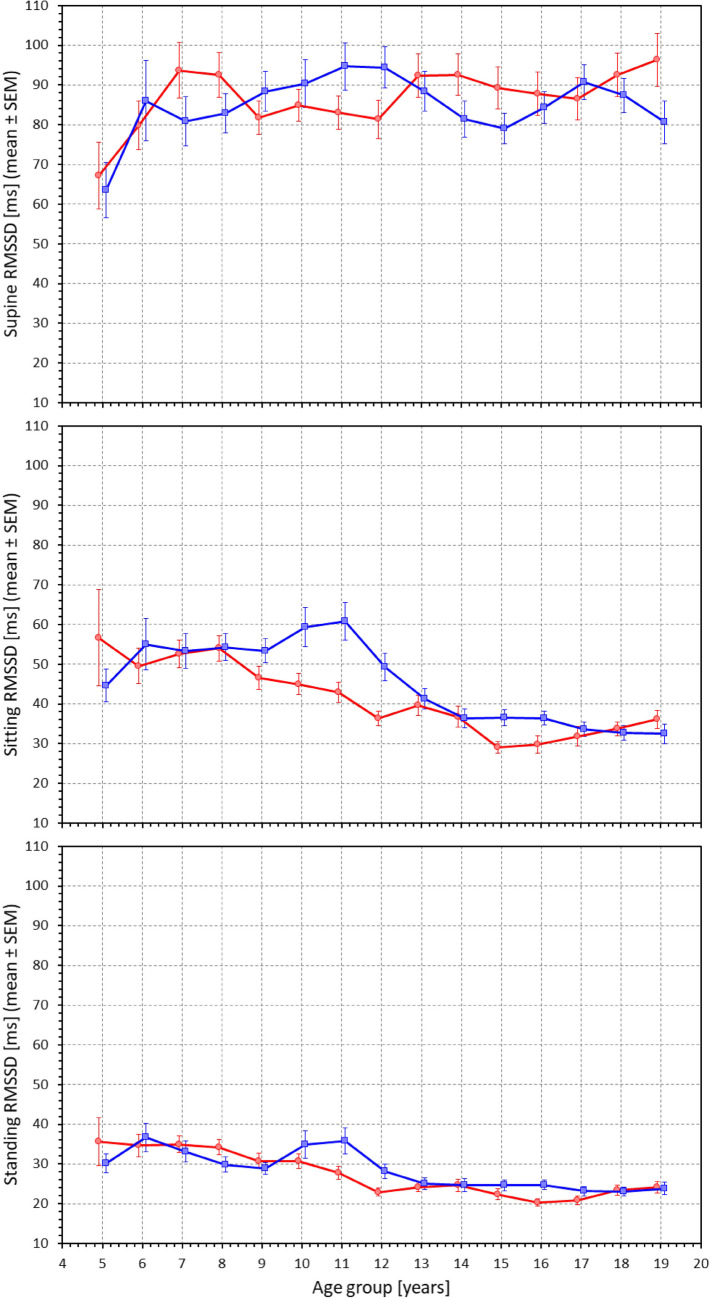
RMSSD values measured in 2-year bins of the population in supine position (top panel), sitting position (middle panel) and standing position (bottom panel). The layout of the figure, definition of population strata, and distinction between females and males are the same as in Fig. 3. RMSSD – root mean square of successive differences of RR intervals, SEM – standard error of mean

**Fig. 7:**
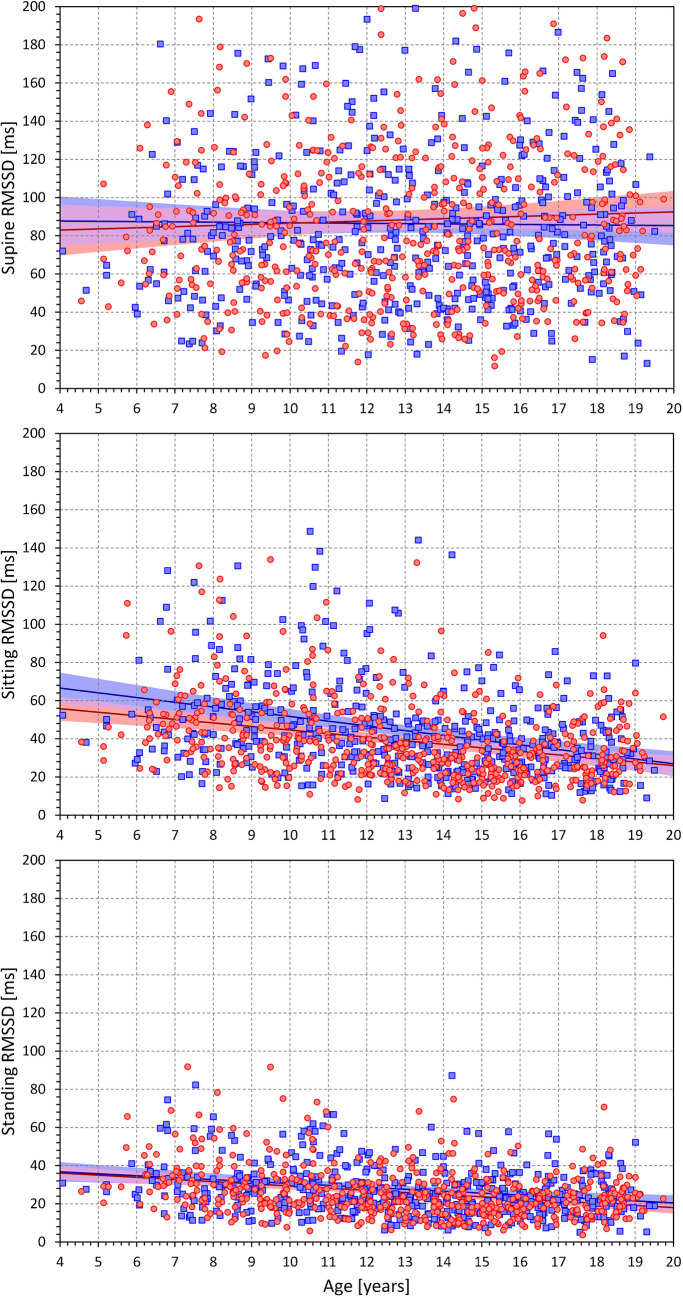
Scatter diagrams between the ages of study subjects and their RMSSD values measured in supine position (top panel), sitting position (middle panel) and standing positions (bottom panel). The layout of the figure and the meaning of the symbols, lines, and bands is the same as in Fig. 5. RMSSD – root mean square of successive differences of RR intervals

Figure 6 shows the age-related development of RMSSD values measured at different postural positions. Somewhat unexpectedly, the figure is very different from Fig. 3. The comparison of the top panels of Figs. 6 and 3 shows that in children, the relationship between RMSSD value and the underlying heart rate is not present in children recorded at supine rest. Although the supine heart rate was very much higher in small children, the corresponding RMSSD values did not decrease. The supine RMSSD data did not show statistically significant differences between age-defined population quartiles (NS for both females and males). Not surprisingly, gradual decreases in RMSSD, in line with increasing heart rates, were seen in comparisons of sitting and standing positions with the supine position (Supplementary Fig. 1). Nevertheless, these decreases were smallest in the youngest children and gradually increased with the age of the population strata. The independence of the supine RMSSD values of the age of the subjects (and mild dependencies in the sitting and standing positions) are also seen in Fig. 7. The slopes of liner regression of supine RMSSD vs age were 0.60 and -0.15 ms/year in females and males, respectively (both NS). At sitting and standing positions, the corresponding values were -1.86 and -2.48, and -1.13 and -1.02 ms/year, respectively, all p < 0.0001.

The comparison of the SDNN values showed similar results as those obtained with RMSSD and are presented in Supplementary Figs. 2, 3, and 4.

**Fig. 8:**
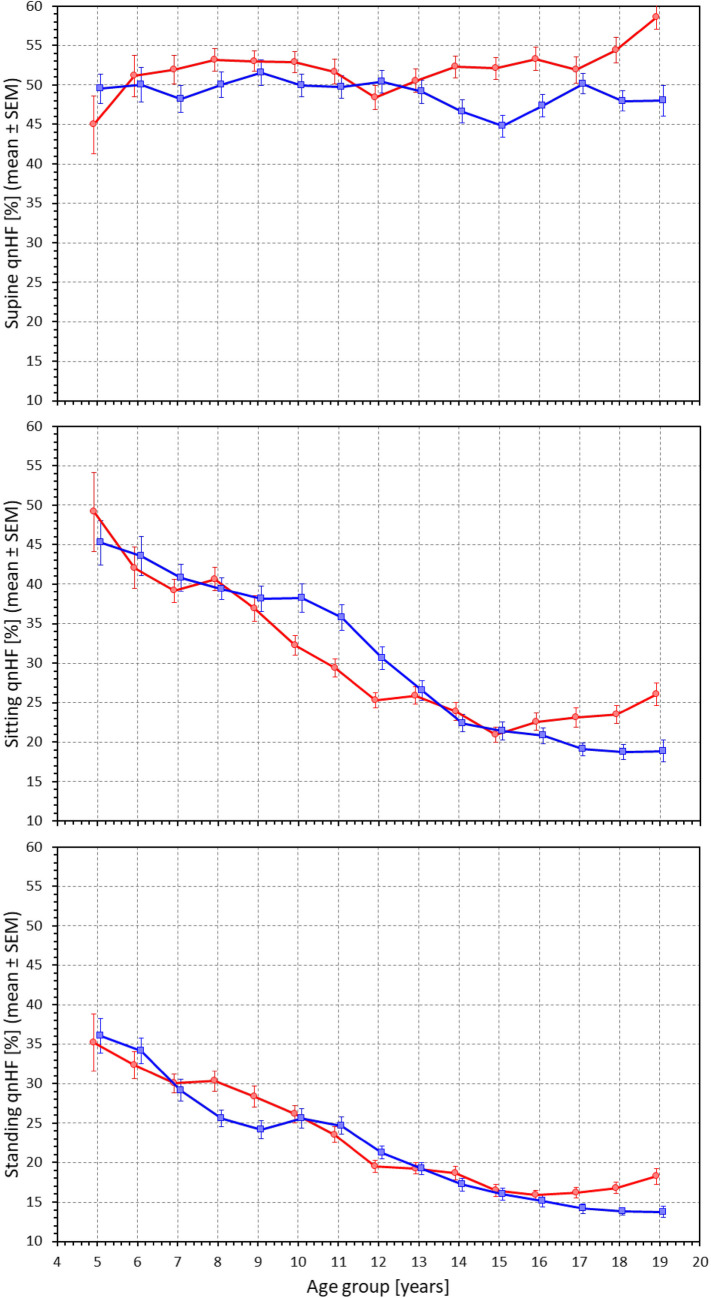
Values of quasi-normalized high frequency heart rate variability components measured in 2-year bins of the population in supine position (top panel), sitting position (middle panel) and standing position (bottom panel). The layout of the figure, definition of population strata, and distinction between females and males are the same as in Fig. 3. qnHF – quasi-normalized high frequency components, SEM – standard error of mean

**Fig. 9:**
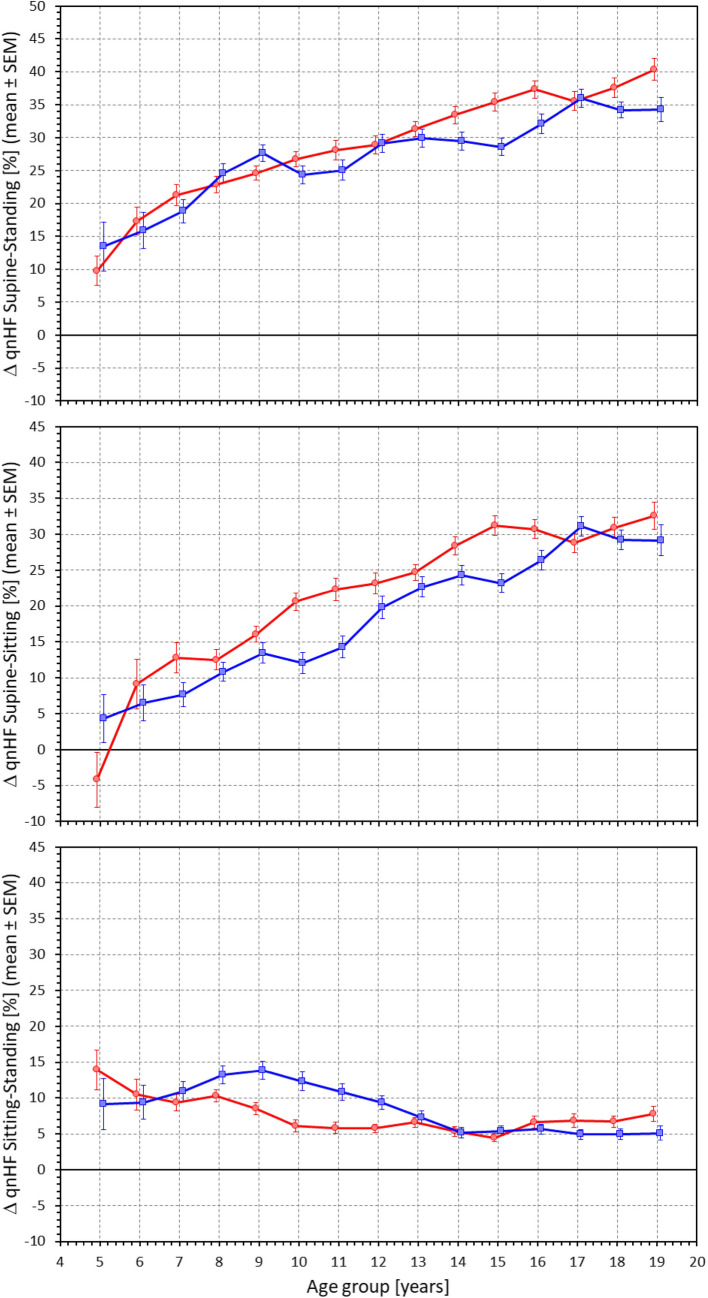
Differences between quasi-normalized high frequency heart rate variability components measured in standing and supine positions (top panel), sitting and supine positions (middle panel) and standing and sitting positions (bottom panel). The layout of the figure, definition of population strata, and distinction between females and males are the same as in Fig. 3. ΔqnHF – differences in quasi-normalized high frequency components, SEM – standard error of mean

**Fig. 10:**
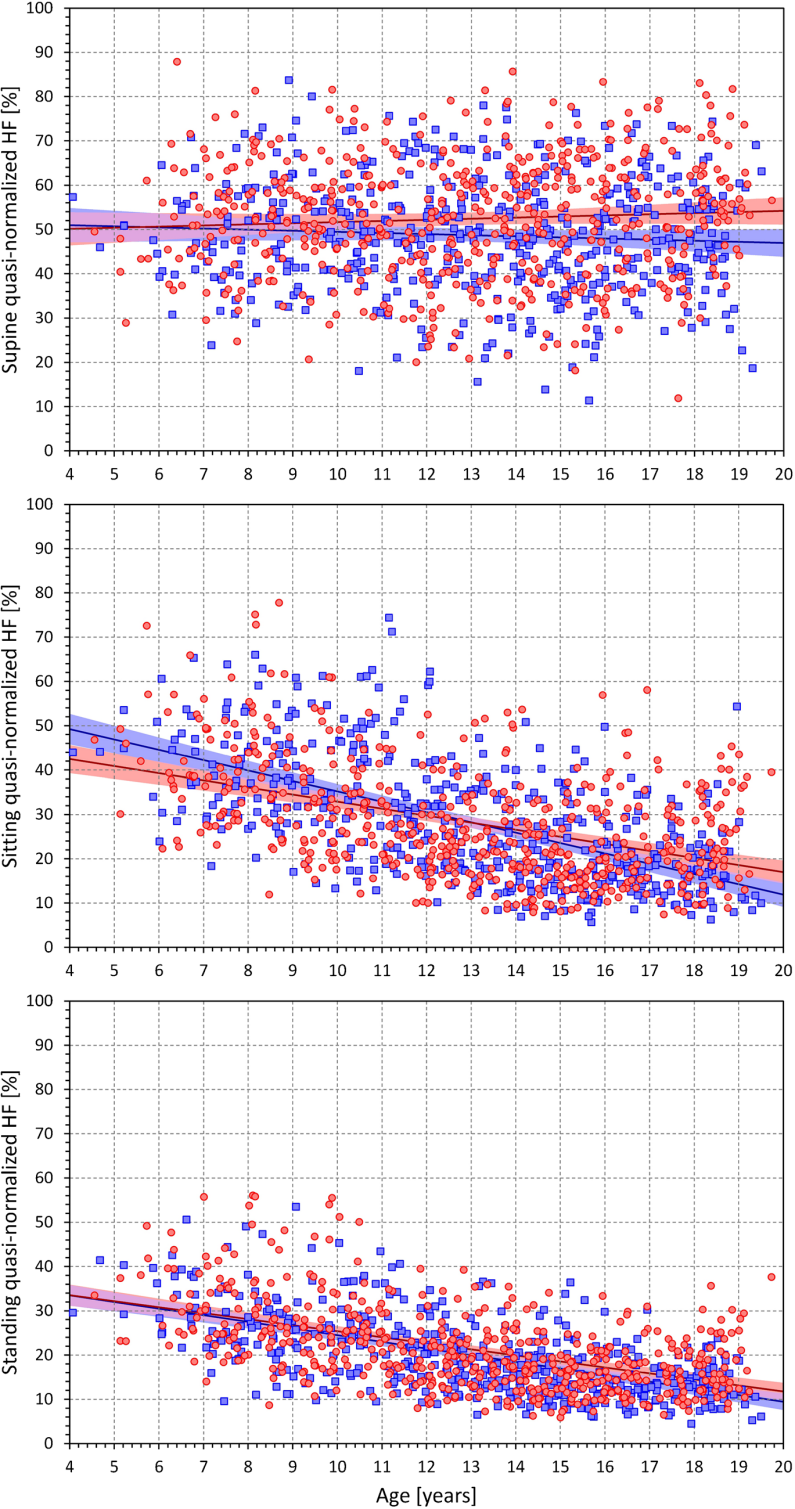
Scatter diagrams between the ages of study subjects and their quasi-normalized high frequency heart rate variability components measured in supine position (top panel), sitting position (middle panel) and standing position (bottom panel). The layout of the figure and the meaning of the symbols, lines, and bands is the same as in Fig. 5. HF – high frequency components

Figure 8 shows the age-related development of HRV quasi-normalized HF components measured at different postural positions. Figure 9 shows the differences in these components between the postural positions. To a substantial extent, the results replicate the observations that have been made with RMSSD values. In supine position, there was little age influence on the components with the exception of a marginal decrease in males noted from approximately the age of 12 years. Indeed, comparison between age-related population quartiles was not statistically significant in females but statistically significant in males (p = 0.007). Compared to supine position, the components decreased in sitting and standing positions with the decrease noticeably increasing with increasing age. These observations were also visible in the scatter diagrams displaying individual values of quasi-normalized HF components (Fig. 10). The regression slopes of the components were much shallower in supine position (0.253 and -0.250% per year in females and males, respectively, both NS) compared to sitting (-1.592 and -2.334% per year in females and males, respectively, both p < 0.0001) and standing (-1.361 and -1.503% per year in females and males, both p < 0.0001).

**Fig. 11:**
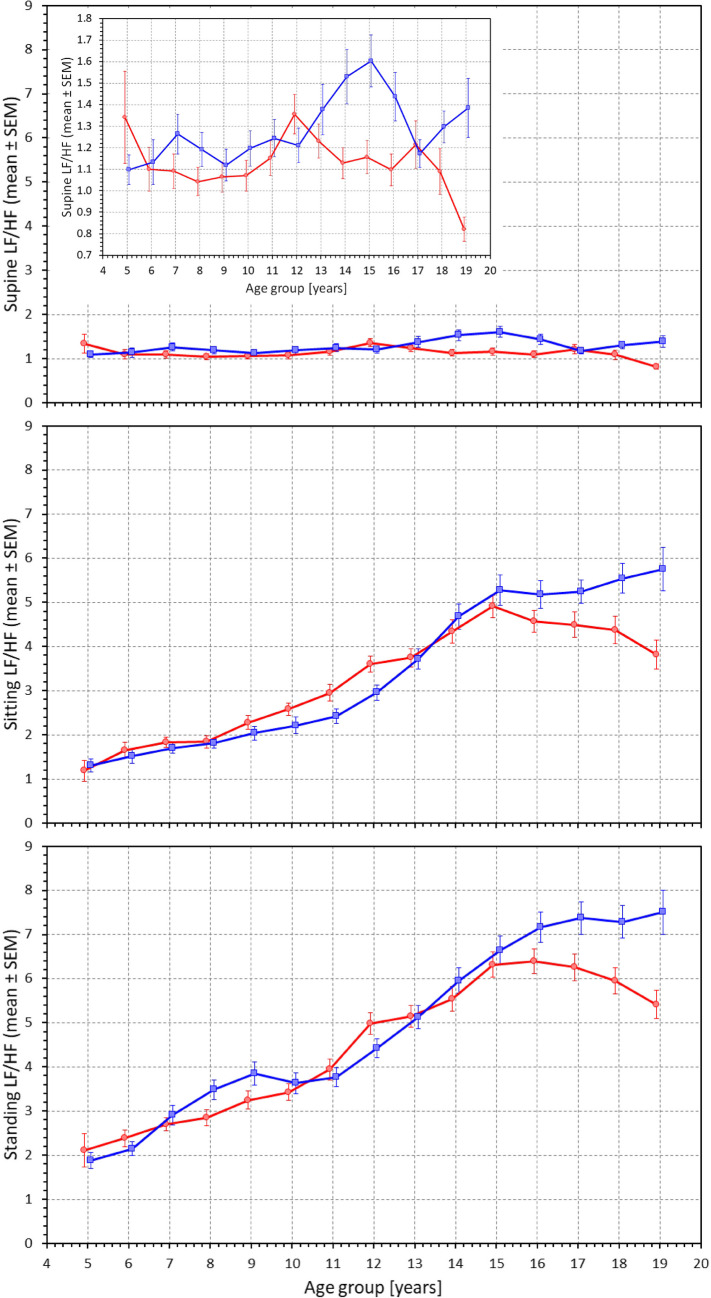
Values of proportions between low frequency and high frequency heart rate variability components measured in 2-year bins of the population in supine position (top panel), sitting position (middle panel) and standing position (bottom panel). The layout of the figure, definition of population strata, and distinction between females and males are the same as in Fig. 3. The sub-graph in the top panel shows the same data on magnified vertical axis. LF/HF – proportions between low frequency and high frequency components, SEM – standard error of mean

Consistent results have also been found when studying the age-dependence of LF/HF proportion (Fig. 11 and Supplementary Figs. 5 and 6). In supine position, there was little albeit statistically significant difference between age-defined population quartiles in males (p = 0.0001) which was not present in females (NS). In males, marginal increases in LF/HF are seen in supine position from the age of approximately 12 years. In supine position, the slopes between LF/HF value and age were shallow (-0.003 per year in females, NS; + 0.023 per year in males, p = 0.0423). In sitting and standing positions, the dependency of LF/HF proportions on age showed clearly positive regression slopes (0.256 and 0.420 per year in females and males in sitting position, and 0.334 and 0.472 per year in females and males in standing position, all p < 0.0001).

## Discussion

The data of the study lead to potentially novel considerations in respect of the development of cardiac autonomic status in children and adolescents. Direct translation of HRV measures into values of vagal and sympathetic tone is problematic [34, 35]. Nevertheless, it is reasonable to suggest and consequently also widely accepted [62] that increase and decrease of short-term high-frequency modulations of RR periods indicate an increase and decrease in cardiac vagal influence. Long-frequency RR modulations inform about both autonomic limbs but if taken relative to the high-frequency modulations (as in the LF/HF ratio) approximation of sympathetic influence becomes reasonable.

In younger and middle-aged adults, the dependency between HRV indices and the underlying heart rate has been intensively studied [9, 42, 53, 54]. In general, increased heart rate leads to decreased numerical values of HRV indices for both mathematical and physiologic reasons. In older adults, variability of heart rate periods is known to decrease and disassociation between HRV changes and heart rate changes with advancing age have been reported [8]. Observations corresponding to younger adults were also made in paediatric recordings [39]. It was therefore somewhat surprising that we found the supine RMSSD values (and similarly the supine quasi-normalized HF components and LF/HF ratios) practically independent of age, although the underlying heart rate was very much dependent on age. In undisturbed stable and physiologic conditions, both RMSSD values and HF components reflect mainly sinus arrhythmia which is mostly, although not entirely, a consequence of cardiac period modulations due to respiration. This is, in turn, understood to be caused primarily by vagal reactions [7, 35, 36, 62]. Our observations made during supine conditions therefore contradict the conjecture that vagal control of heart rate is underdeveloped in small school-age children and gradually matures only around puberty. Indeed, if we applied proposed technical controls for the underlying heart rate [4, 15] “corrected” supine RMSSD and HF values would be larger in smaller children compared to adolescents.

We also observed that the supine → sitting and similarly supine → standing changes had smaller effects in little children, both in terms of heart rate elevation, LF/HF increase, and RMSSD and quasi-normalized HF reduction. Using the same model of cardiac autonomic control, this might be interpreted as the vagal heart rate modulations being less reduced in small children during provocative manoeuvres that lead, in older children to vagal suppression by increased sympathetic influence, consistent with observations repeatedly made in adults [24, 47].

A model different to increased sympathetic and reduced vagal influence of heart rate therefore needs to be proposed to explain the elevated heart rate in small children. We note that changes from supine to standing position led to heart rate and LF/HF increases as well as RMSSD and quasi-normalized HF decreases that were much larger in older compared to younger children. Both heart rate and the HRV indices measured in standing position consequently differed across the population less than when measured in supine position. In adults, change from supine to standing position leads to sympathetic charge that also suppresses vagal heart rate influence while the opposite change reduces sympathetic influence and boosts vagal heart rate modulations [35, 36, 47]. Considering these mechanisms, it seems plausible to propose that autonomic heart rate control is less balanced in young children with sympathetic branch less responsive to vagal influence.

In other words, it appears that the different facets of our observation would be reasonably explained by gradual age-related increases of sympathetic responsiveness to the parasympathetic changes. By this we mean that the ability of sympathetic system to increase heart rate is present in both small and older children. While the variable vagal influence is able to modulate cardiac periods in both young and older children equally, it is less able to suppress the sympathetic influence in younger children. Nevertheless, modifications of the balance of vagal and sympathetic modulations might be equally compatible with our results. Such balance modifications have previously been observed in responses to provocation, e.g., during gradual head-up tilt when little HRV change were observed despite obvious heart rate increases [48, 49]. Thus postural challenges might influence parasympathetic and sympathetic modulations in an opposite way.

Different parts of our data are in good agreement with previously reported observations. Dollar et al.[11] performed repeated investigations in 270 children and found little changes in resting respiratory arrhythmia between the ages corresponding to the age span of our population. Gatzke-Kopp and Ram [16] subjected 339 children to different psychological tasks and described decrease in respiratory arrhythmia with age, reminiscent of our observations during sitting. Moderate decrease of respiratory arrhythmia over the ages 8 to 10 years was also reported in a smaller population of Caucasian children investigated in sitting positions [22, 41]. Numerically, the supine and standing RMSSD values that we observed were comparable to those reported in a smaller population by Longin et al.[32] Similar to other HRV studies, we present here the interpretation of the data in terms of autonomic influence. Other possibilities also exist, e.g., direct influence on the electrophysiology (rather than neural regulation) of sinus nodal pacemaker cells or of the atrio-ventricular nodal conduction. In the absence of invasive electrophysiology studies, we are unable to comment on these alternative mechanisms.

Our study also demonstrates the long-discussed differences between physiologic background of heart rate and of HRV. While it is undisputable that for simple mathematical reasons, the numerical values of HRV indices are influenced by underlying heart rate [53, 54], especially if intra-individual changes are considered, the notion that HRV does not offer more physiologic information than the underlying heart rate [5] needs to be refuted [35, 36]. Indeed, our data demonstrate the disassociating between heart rate and HRV very clearly. For instance, our Figs. 3 and 5 show that supine heart rate was substantially decreasing with the age of the subjects while Figs. 6 and 7 show that supine RMSSD values were practically independent of the age, similar to qnHF values (Figs. 8 and 10).

The knowledge of the physiologic development of the cardiac autonomic status in children is of importance for the discrimination between normal physiology and disease by the means of autonomic assessment. While our mechanistic proposal on the development of sympathetic responsiveness appears to explain our measurements satisfactorily, it is difficult to compare it with other studies since limited knowledge exists based on carefully conducted autonomic provocations in children. Some of the previously proposed suggestions of children’s autonomic development do not agree with our data and observations. Harteveld et al.[21] recently reported data from a combination of 5 different paediatric studies that included healthy cohorts of different ages. Analysing ECG investigations performed in sitting position, they report a cubic trend of parasympathetic activity, with an exponential increase from infancy, a plateau phase during middle childhood, followed by a decrease to adolescence. Our baseline supine data do not support such a development although we have observed similar decreases in sitting and standing positions. Nevertheless, as already explained, the differences between supine and autonomically provocative positions that we observed do not allow us to agree with the suggestion that parasympathetic system decreases during late childhood. We attribute the observations to the maturation of sympatho-vagal balance. Harteveld et al.[21] also investigated (still in sitting positions) pre‐ejection periods and describe a gradual decrease in sympathetic activity from infancy to adolescence. Although our data are limited to ECG-derived cardiac periods, the age-related increases in LF/HF ratios that we note in supine and sitting positions do not agree with a gradual decrease of sympathetic activity with advancing age.

Contrary to our expectations [6, 31, 60], we observed only small heart rate differences between girls and boys (note that the regression analysis of the supine data suggested sex differences of females minus males increasing by 0.13 bpm per year). This might have been contributed by the differences in athletic training in the post-pubertal parts of our data. The differences in the age-relationship slopes of supine quasi-normalized HF components and LF/HF ratios appear to agree with the previously made observations that female adults at rest show higher vagal modulations of heart rate compared to males [1, 12, 24, 52, 59]. Combined with the distribution of secondary sex signs in the population, our observations offer an explanation that this difference is caused by post-pubertal increases in sympathetic modulation in males. Because of the lack of hormone measurements, we are unable to answer the obvious question of whether this is caused by testosterone effects on the maturation of sympatho-vagal balance.

## Limitations

A number of limitations of our study also need to be considered. The study procedures were limited to ECG recordings. Since we have not performed direct measurements of vagal and sympathetic tone and nerve traffic, we cannot distinguish between vagal ability to suppress sympathetic influence (e.g., in younger children) and increased ability of sympathetic control to impose a vagal withdrawal (e.g., in older children and adolescents). We have also concentrated on HRV indices with well recognised interpretation in terms of autonomic control. Different elaborations of our data are needed to study age-related development of other indices such as symbolic dynamics of RR intervals [64, 65], HRV entropy estimates [48], fractal properties of RR tachograms [33], and their detrended fluctuation analysis [45].For organizational as well as limited support reasons, we were unable to collect additional measurements such as continuous blood pressure signals and beat-to-beat pre‐ejection periods. We therefore cannot support our findings by independent analyses, e.g. by non-invasive assessment of baroreflex sensitivity [3]. For the same reasons of study practicality and limited support, we were also unable to record respiration [14]. We attempted to approximate respiration frequency based on ECG morphology using the algorithms validated in supine adults [57] but these attempts were unsuccessful especially in the sitting and standing positions, and in young children. Our data permit studying short-term beat-to-beat QT interval variability [51] but this was omitted in the present investigation. Although we collected demographic data, the analyses of the study did not consider relationship of cardiac autonomic balance to body mass index [56] or to level of athletic training [2]. For ethical reasons, we were not able to collect blood samples and thus, our assessment of puberty was strictly based on the secondary sex signs. Together with their appearance, we have also collected data on the last menstruation in post-puberty females but have not used this information in the analyses presented in this text [29]. The population of the study enrolment area does not contain sufficient number of minorities and therefore, the investigated population was composed mostly of Caucasian subjects which prevented any statistically viable race comparisons [19]. Figure 1 shows that heart rate levels were not fully reproducible in repeated postural positions. We assume that this was caused by sequential effects of the protocol but have not studied sequential effects of the HRV measurements [26]. Practical and support restrictions allowed to investigate each subject only once. Therefore, we cannot comment on any reproducibility of the protocol which would need to be assessed before proposing it for the detection of autonomopathies [38]. The study also did not include any children or adolescents with previously diagnosed autonomic abnormalities such as idiopathic autonomic conditions [18, 55] and we thus cannot comment on possible diagnostic use of the protocol.

## Conclusion

In spite of all the limitations, the results obtained in the study allow us to propose that during childhood and adolescence, the maturation of the cardiac autonomic status manifests mainly in the increased responsiveness of the sympathetic nervous influences. The extent of short-term beat-to-beat changes in the duration of cardiac periods was little influenced when assessed in supine position, i.e., under conditions of little sympathetic induced stress. In sitting and standing positions, the short-term beat-to-beat variability of cardiac cycles decreased with increasing age suggesting progressive maturation of sympathetic responsiveness. The study also proposes that the increased heart rate in small age children is caused by sympathetic drive that lacks the ability to respond to the vagal influence.

### Supplementary Information

Below is the link to the electronic supplementary material.

Supplementary file1 (PDF 1575 KB)

## Data Availability

The data supporting the conclusions of this article will be made available by the authors pending the approval by the Ethics Committee of University Hospital Brno and by the Steering Committee of the main project of paediatric data collection. Requests for the data are to be sent to the corresponding author together with a detailed plan of the proposed analyses.
