# Supplementary material for: Development of autonomic heart rate modulations during childhood and adolescence
Source: Pflugers Arch. 2024 Jun 28;476(8):1187–207. doi: 10.1007/s00424-024-02979-0 (PMC11271370; doi:10.1007/s00424-024-02979-0)

Development of autonomic heart rate modulations during childhood and adolescence

by

Kateřina Helánová[1,2], Martina Šišáková[1,2], Katerina Hnatkova[3],
Tomáš Novotný[1,2], Irena Andršová[1,2], Marek Malik[3,2]

[1] Department of Internal Medicine and Cardiology, University Hospital Brno,
Brno, Czech Republic

[2] Department of Internal Medicine and Cardiology, Faculty of Medicine,
Masaryk University, Brno, Czech Republic

[3] National Heart and Lung Institute, Imperial College,
London, England

Supplementary figures

Supplementary figures have the same layout and symbol meaning as the corresponding style figures of the main manuscript.

Supplementary

Figure S1

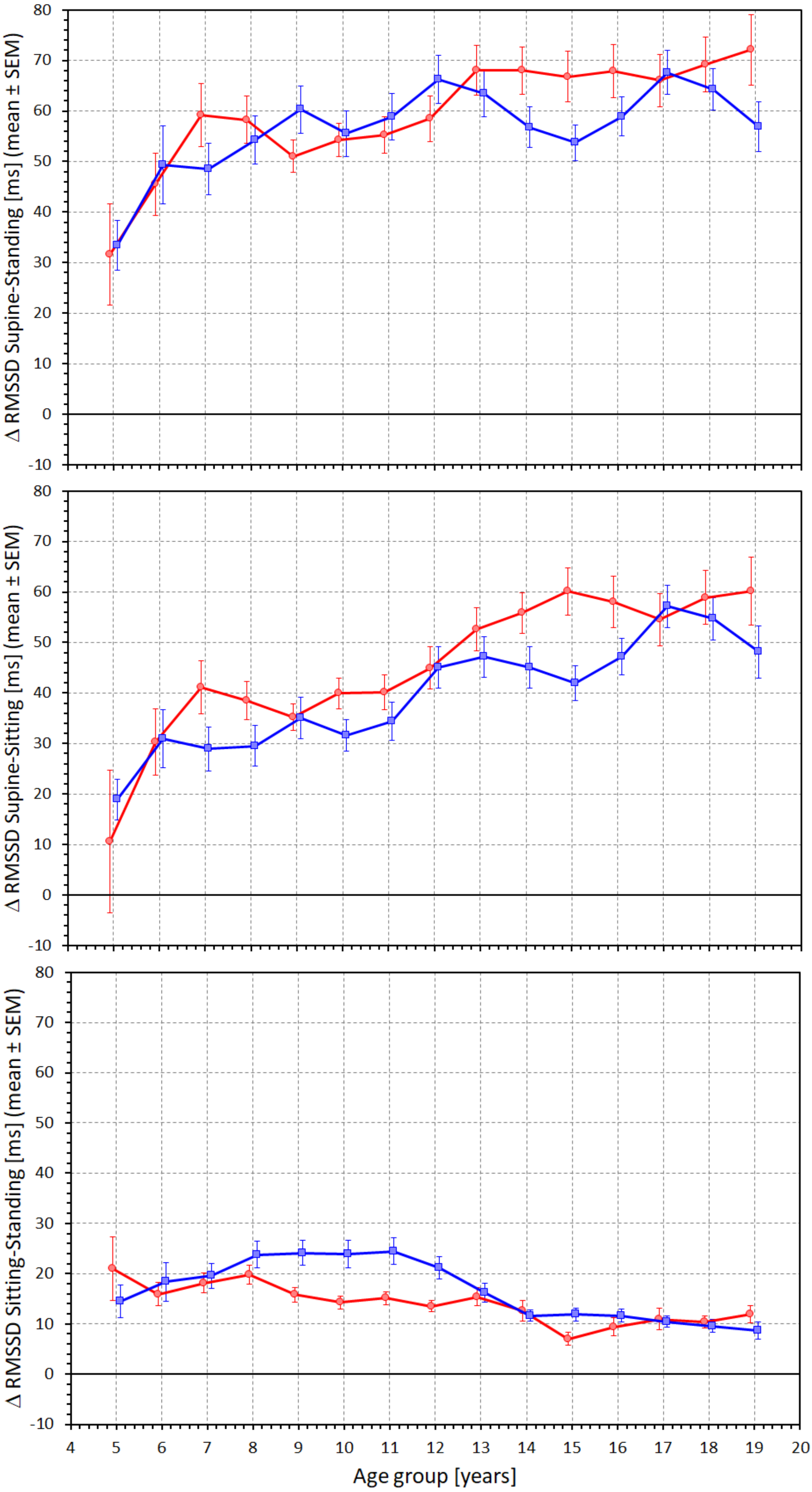

Supplementary

Figure S2

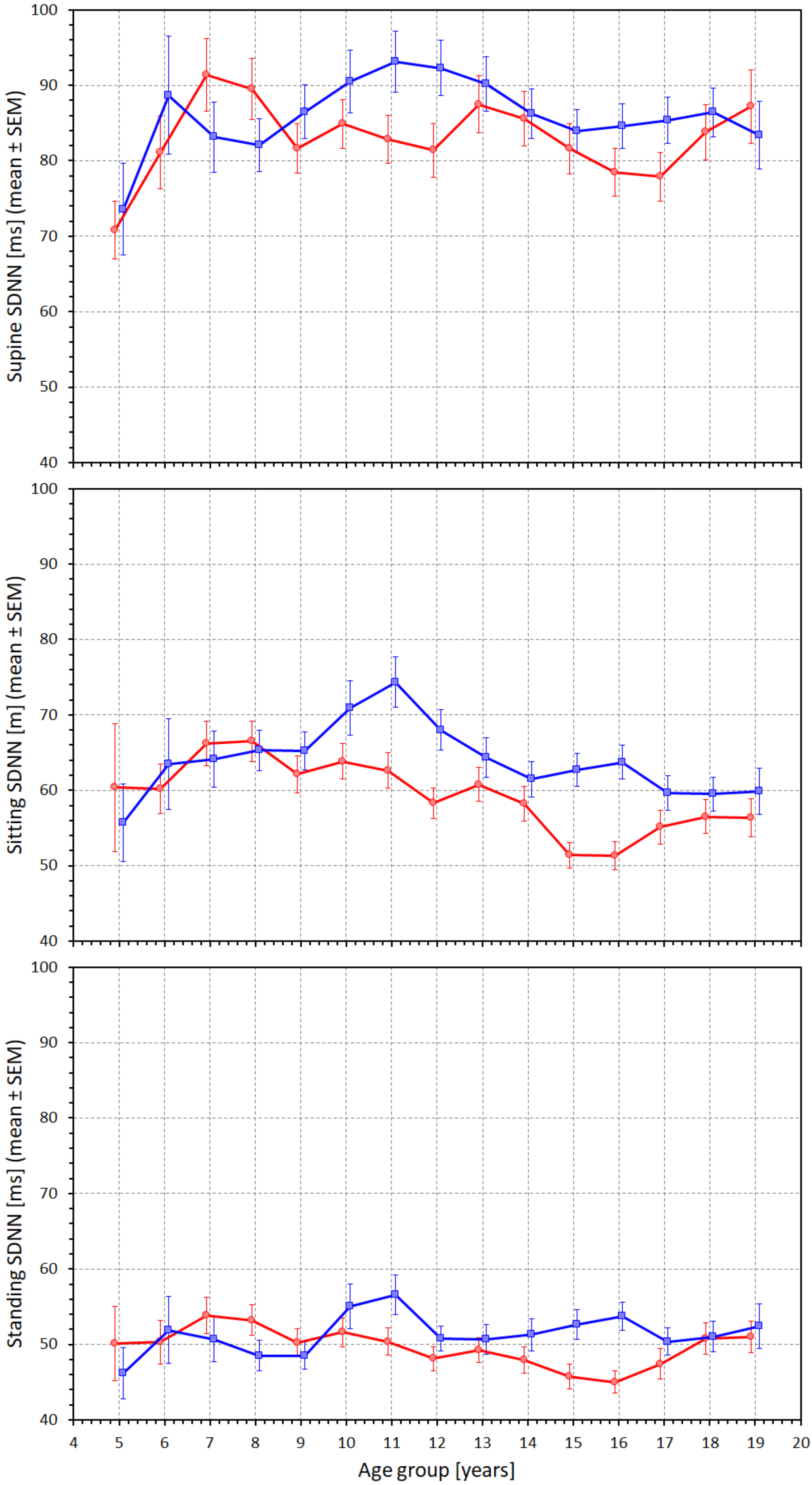

Supplementary

Figure S3

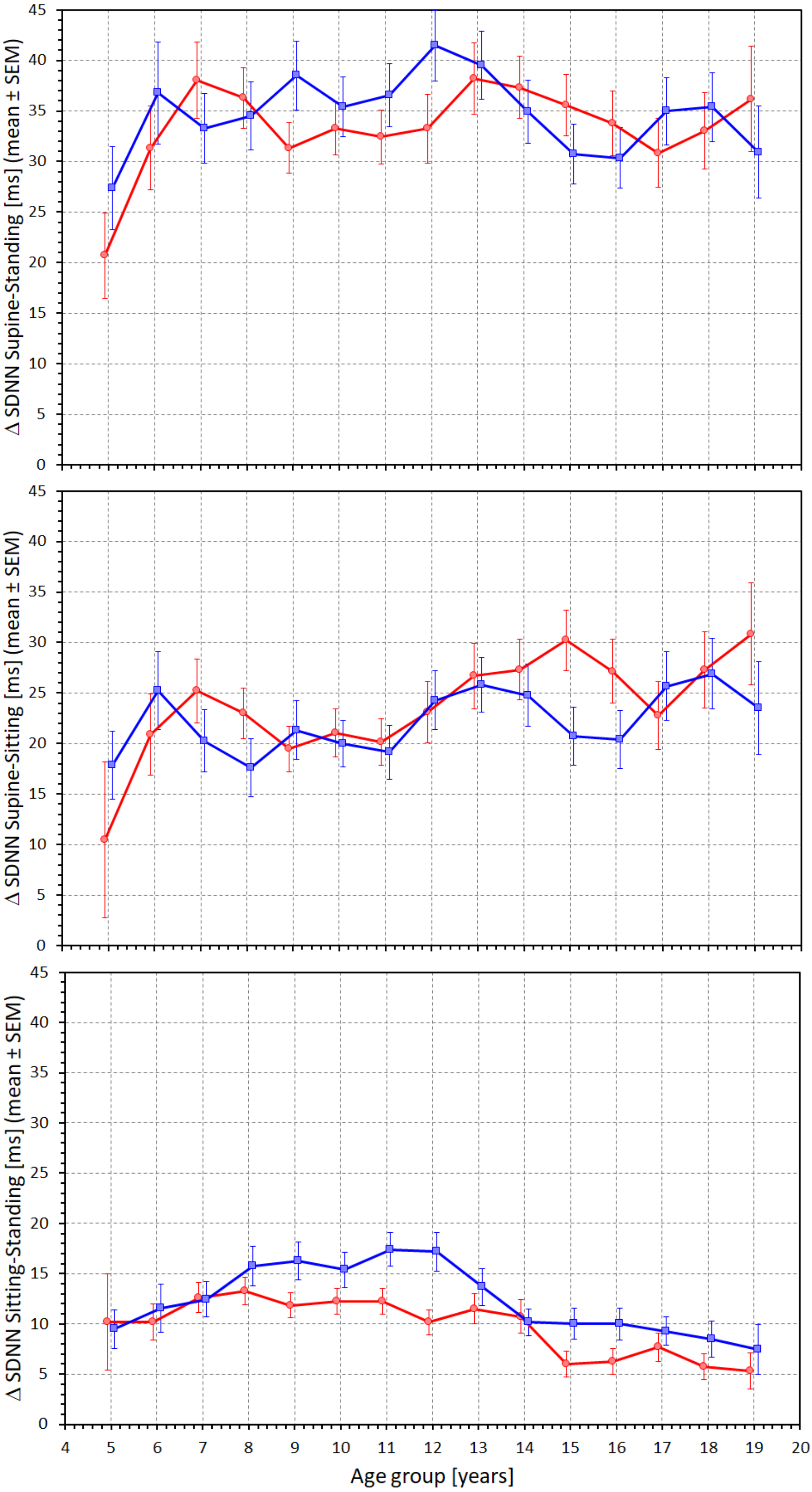

Supplementary

Figure S4

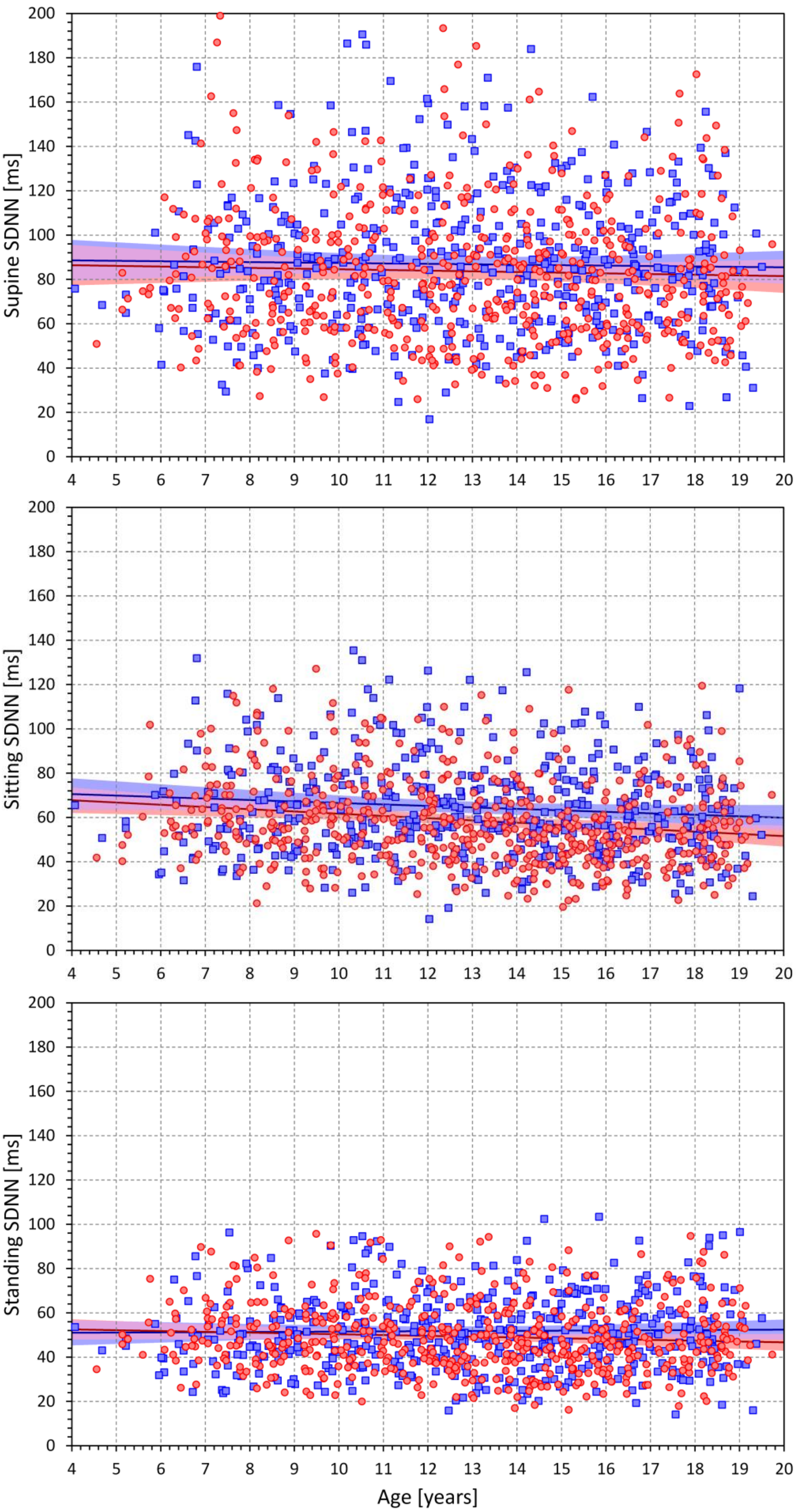

Supplementary

Figure S5

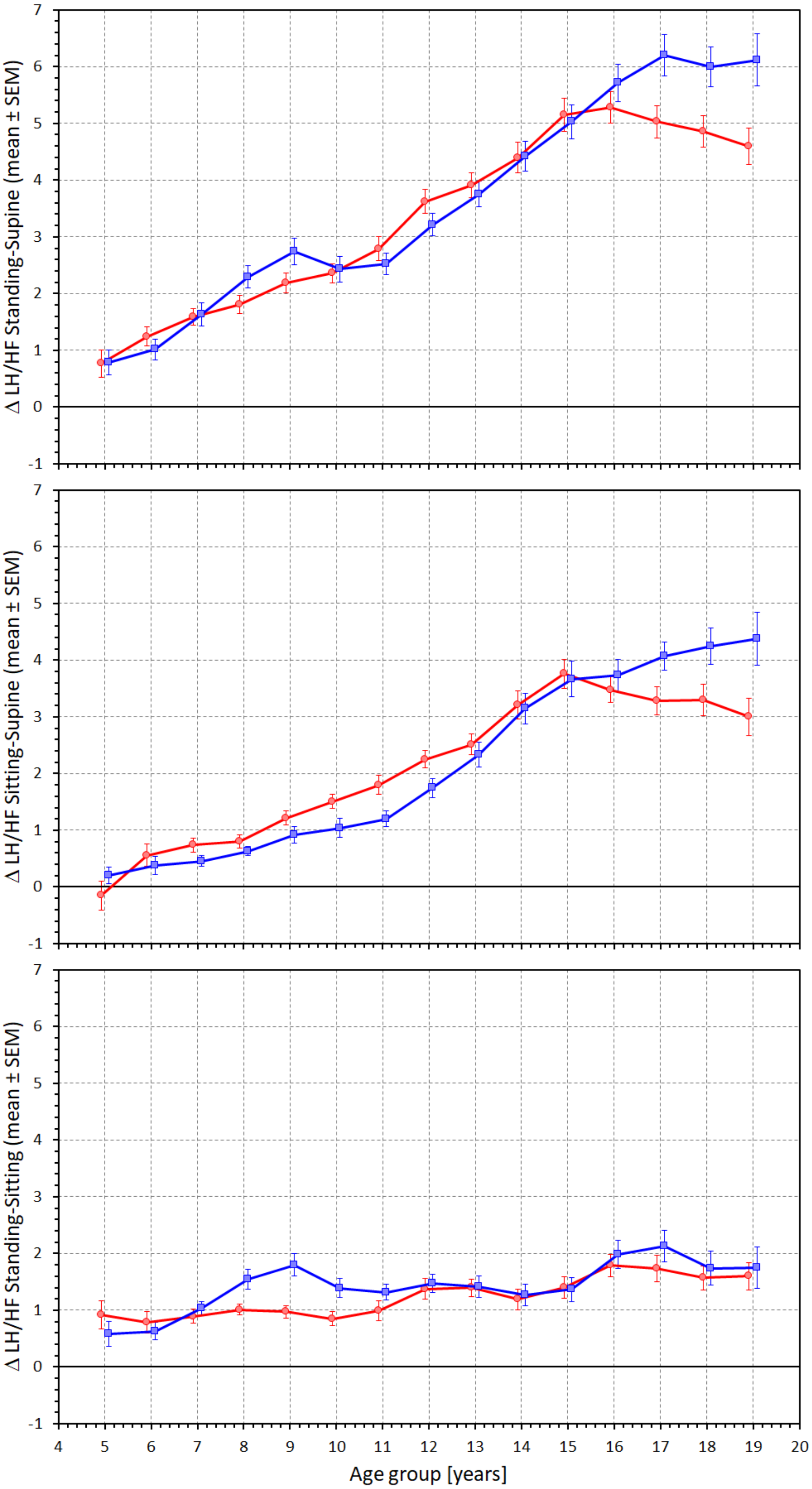

Supplementary

Figure S6

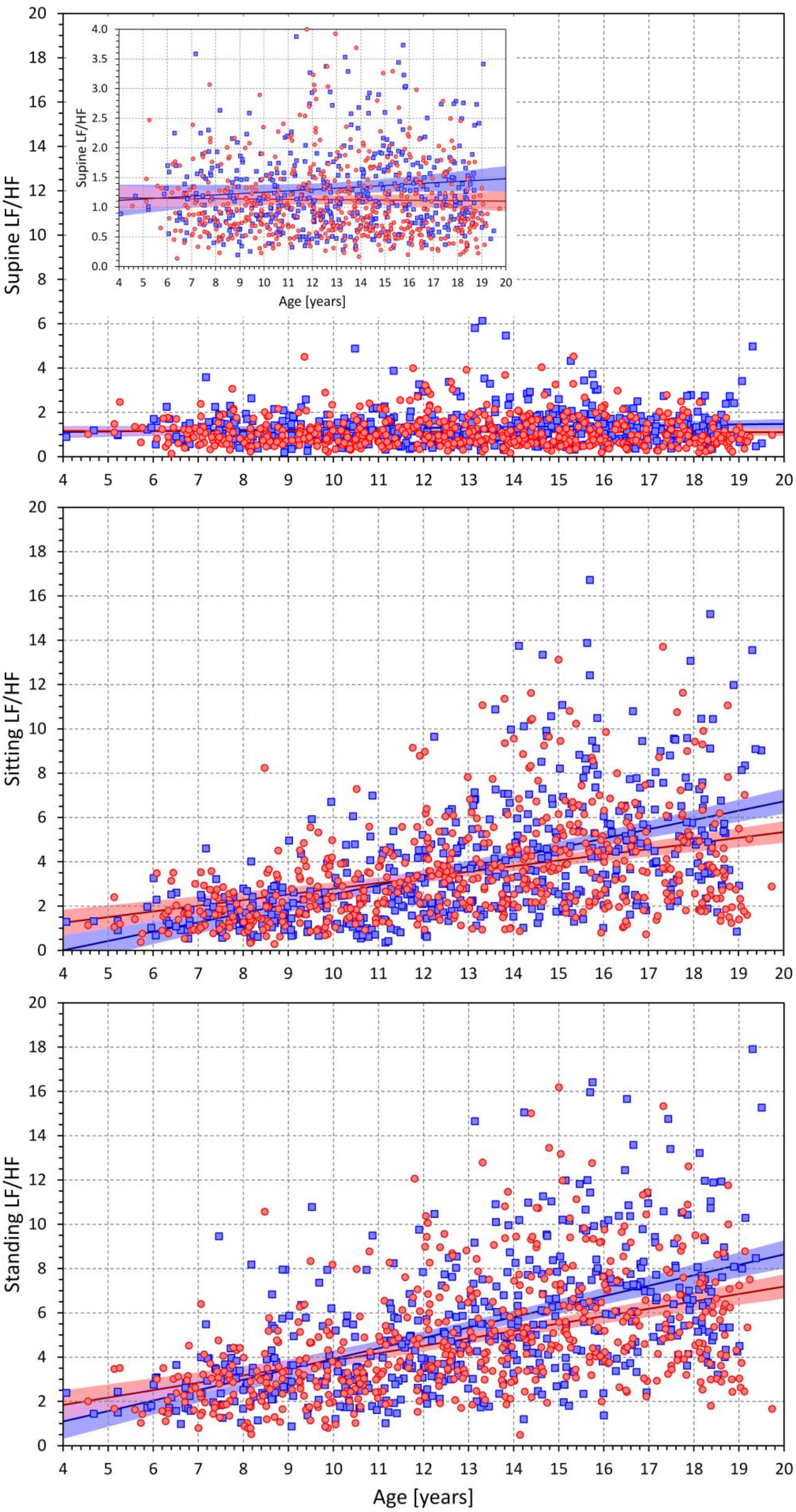

Supplement: Supplementary file 1 — Supplementary file1 (PDF 1575 KB) [file 424_2024_2979_MOESM1_ESM.pdf]
